# Pulmonary Presentation of Kaposi-Sarcoma in a Renal Transplant Recipient

**DOI:** 10.7759/cureus.6719

**Published:** 2020-01-21

**Authors:** Seth Scheetz, Deepali Pandey, Todd E Pesavento, Priyamvada Singh

**Affiliations:** 1 Internal Medicine, The Ohio State University College of Medicine, Columbus, USA; 2 Internal Medicine, Saint Vincent Hospital, Worcester, USA; 3 Nephrology and Comprehensive Transplant Center, The Ohio State University Wexner Medical Center, Columbus, USA

**Keywords:** kaposi sarcoma, renal transplant, hhv-8

## Abstract

Renal transplant patients on immunosuppression are at risk for malignancy. One form of malignancy that commonly affects this population is Kaposi-sarcoma. Kaposi-sarcoma is a human herpesvirus-8 (HHV-8)-driven process classically associated with skin lesions in immunocompromised patients. The pulmonary system may be involved in disseminated disease. In this case, a renal transplant patient was re-admitted with acute hypoxic respiratory failure and hemoptysis of an unclear etiology. Following a broad workup, HHV-8 PCR and a lymph node biopsy confirmed pulmonary Kaposi-sarcoma. Workup for multicentric Castleman disease was negative. The patient was treated with liposomal doxorubicin, ganciclovir, and prednisone. Her immunosuppression was changed to sirolimus and she is scheduled to complete six cycles of liposomal doxorubicin.

## Introduction

Solid organ transplant recipients on immunosuppression are at increased risk for malignancy [1]. Specifically, renal transplant patients are about three times more likely to develop cancer, including skin cancer, lip cancer, post-transplant lymphoproliferative disease, anogenital cancer, renal cell carcinoma, and Kaposi-sarcoma. Malignancy may present with nonspecific signs including thrombocytopenia and generalized lymphadenopathy. These signs, however, may also be seen in autoimmune and infectious processes, making diagnosis challenging. We present here a diagnostic dilemma of a case of a pulmonary presentation of Kaposi-sarcoma (KS) in a kidney transplant recipient.

## Case presentation

**Figure 1:**
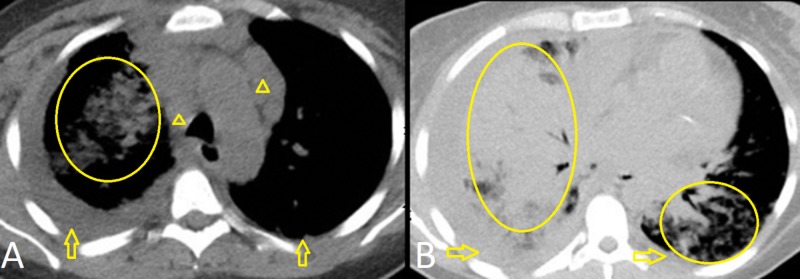
Chest CT of upper lungs (A) and lower lungs (B) showing multifocal bronchopneumonia (oval), moderate right and small left partially loculated pleural effusions (arrow), and enlarged mediastinal and bilateral hilar lymph nodes (arrowhead). Bilateral axillary and supraclavicular lymph nodes were also enlarged.

**Figure 2:**
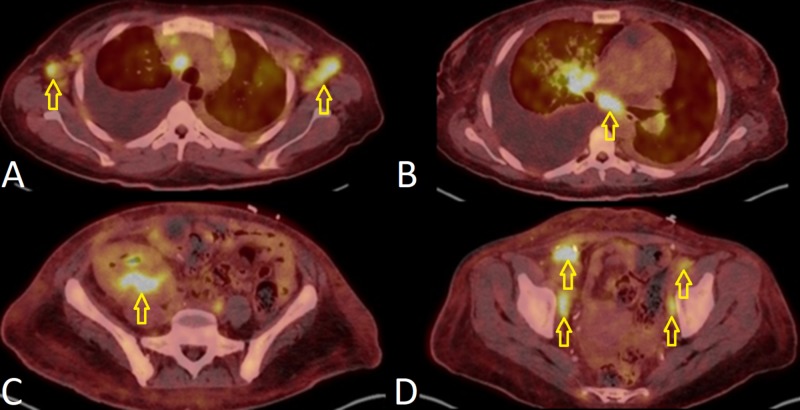
Positron emission tomography (PET) scan for staging of Kaposi-sarcoma. Bilateral axillary lymphadenopathy (A), subcarinal lymphadenopathy (B), uptake in the transplanted kidney (C), bilateral inguinal lymphadenopathy (D).

**Figure 3:**
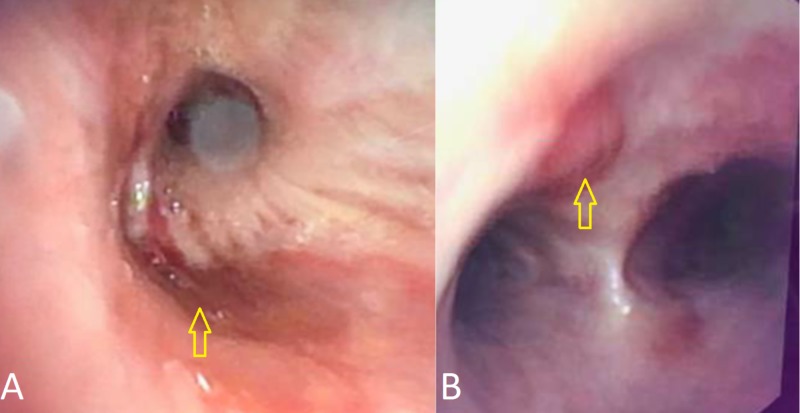
Bronchoscopy showing erythematous patches (A) and purpuric lesions (B) in the airways consistent with Kaposi-sarcoma.

A 43-year-old female with end-stage renal disease secondary to type two diabetes mellitus, status-post renal transplant one year prior, on tacrolimus and myfortic, was admitted with acute hypoxic respiratory failure (new oxygen requirement of eight liters per minute). She had two similar admissions within two months and was treated for community-acquired pneumonia and volume overload. CT scan during those admissions showed bilateral nodular infiltrates, diffuse lymphadenopathy (hilar, mediastinal, inguinal, and axillary), and moderate pleural and pericardial effusions. She was discharged with plans for an outpatient lymph node biopsy if an eight-week follow-up CT-chest did not show improvement. During this encounter, her respiratory status worsened to require high-flow-oxygen and she developed hemoptysis. Her initial CT scan presentation was similar (Figure 1A, 1B). An extensive workup was negative for infectious (pan-culture, immunocompromised respiratory panel, Epstein-Barr virus [EBV], cytomegalovirus [CMV], BK, Pneumocystis jiroveci pneumonia [PJP], adenovirus, fungitell, HIV, streptococcus pneumonia, and legionella) and autoimmune (antinuclear antibody [ANA], double stranded DNA [dsDNA], antineutrophil cytoplasmic antibody [ANCA], anti-glomerular basement membrane [GBM]) etiologies. She was non-responsive to diuretics and broad-spectrum antibiotics. A thoracentesis and bronchoscopy were consistent with an exudative process, narrowing the differential to autoimmune, infectious, or malignant processes with concern for diffuse alveolar hemorrhage (DAH). An axillary lymph node biopsy showed HHV-8+ KS. Further history revealed one month of a violaceous skin rash and gingival lesion. HHV-8 polymerase chain reaction (PCR) quantity was 87,572. A positron emission tomography (PET) scan for staging showed extensive lymphadenopathy (Figure 2A-2D) [2]. Workup for multicentric Castleman disease (MCD) and hemophagocytic lymphohistiocytosis (HLH) was negative. Endobronchial ultrasound-guided biopsy of a hypermetabolic subcarinal lymph node showed a spindle cell tumor consistent with Kaposi-sarcoma but not multicentric Castleman disease. Bronchoscopy at this time showed lesions consistent with pulmonary KS (Figure 3A, 3B) [3]. We treated her with liposomal doxorubicin, ganciclovir, and prednisone. We changed the immunosuppression to sirolimus given previous literature showing the benefit of mTOR inhibitors in KS. She responded well to treatment and was weaned off oxygen. She will continue on sirolimus and complete six cycles of liposomal doxorubicin.

## Discussion

Renal transplant patients on immunosuppression are at risk for developing Kaposi-sarcoma [1]. This has been reported to occur as soon as four months post-transplant [4]. The initial presentation classically involves a violaceous skin rash. This rash, however, may be small, hidden, or misdiagnosed, leading to a delay in diagnosis. In this case, a violaceous gingival lesion had previously been documented but not seen as a mucocutaneous sign of malignancy. Kaposi-sarcoma may disseminate to involve visceral organs including the lungs, gastrointestinal tract, lymph nodes, or transplanted kidney [5,6]. Pulmonary Kaposi-sarcoma may be fatal if untreated. In renal transplant patients, Kaposi-sarcoma has been shown to advance rapidly and early staging with a PET scan may expedite diagnosis and initiation of treatment [7].

The patient’s thrombocytopenia, generalized lymphadenopathy, exudative pleural fluid, and bronchoscopic findings were consistent with malignancy, infection, or an autoimmune process presenting as diffuse alveolar hemorrhage. This complicated therapeutic decision making, as management of infection or malignancy is diametrically opposite to the management of an autoimmune process. Malignancy and infection require a reduction of immunosuppression, whereas diffuse alveolar hemorrhage requires intensification of immunosuppression.

In this case, an excisional biopsy earlier in the hospital course may have expedited the diagnosis. During a previous hospitalization, an inpatient lymph node biopsy was not warranted due to improving clinical status and the patient was scheduled for an outpatient lymph node biopsy. During this encounter, however, the inpatient lymph node biopsy was indicated due to generalized lymphadenopathy of unclear etiology and worsening clinical status [8]. A study has shown that inpatient excisional biopsies may lead to quicker diagnoses in hematologic disease, but outpatient ones are more cost-effective [9]. In immunocompromised patients, a high index of suspicion is required to prompt a full skin exam and potentially earlier inclusion of an excisional lymph node biopsy.

The immunosuppression regimen should be reconsidered after a diagnosis of Kaposi-sarcoma in a renal transplant patient. Calcineurin inhibitors may be associated with Kaposi-sarcoma, and the reduction of immunosuppression in renal transplant patients may result in tumor regression. On the other hand, the mTOR inhibitor sirolimus has been shown to prevent the progression of Kaposi-sarcoma in renal transplant patients [10]. The patient’s immunosuppression was changed to sirolimus following this diagnosis.

In HHV-8+ patients, consideration of multicentric Castleman disease is warranted [11]. The treatment for pulmonary Kaposi-sarcoma is liposomal doxorubicin, whereas the treatment for Kaposi-sarcoma associated multicentric Castleman disease is rituximab and liposomal doxorubicin [12,13]. Although the patient had clinical and laboratory findings concerning for multicentric Castleman disease, the diagnostic lymph node biopsy confirmed the presence of HHV-8 but did not show characteristic changes of MCD. As a result, she was treated with liposomal doxorubicin but not rituximab.

## Conclusions

Kaposi-sarcoma often presents as a skin lesion and lymphadenopathy. This case highlights a rare initial presentation of Kaposi-sarcoma with pulmonary symptoms. A high index of suspicion in the immunocompromised population (solid organ transplant, HIV) and early diagnosis can improve survival.
